# Cytokine Dynamics and Herpesvirus Interactions in Pediatric Liver and Kidney Transplant Recipients: The Distinct Behavior of HCMV, HHV6, HHV7 and EBV

**DOI:** 10.3390/v16071067

**Published:** 2024-07-02

**Authors:** Yessica Sánchez-Ponce, Juan Rafael Murillo-Eliosa, Abigail Morales-Sanchez, Ezequiel M. Fuentes-Pananá

**Affiliations:** 1 Research Unit in Virology and Cancer, Children’s Hospital of Mexico Federico Gómez, Mexico City 06720, Mexico; acisseysan@gmail.com (Y.S.-P.); abimor2002@yahoo.com.mx (A.M.-S.); 2 Postgraduate Program in Biological Science, National Autonomous University of Mexico, Mexico City 04510, Mexico; 3 Clinical Pathology Department, Children’s Hospital of Mexico Federico Gómez, Mexico City 06720, Mexico; ralfdd_67@hotmail.es

**Keywords:** transplantation, herpesviruses, EBV, graft-rejection, cytokines

## Abstract

Pediatric solid organ transplant (SOT) recipients face a challenging balance between immunosuppression and graft rejection. While Epstein–Barr Virus (EBV) and cytomegalovirus (HCMV) are known contributors to post-transplant lymphoproliferative disease and graft rejection, respectively, the roles of herpesvirus 6 and 7 (HHV6 and HHV7) and the impact of these herpesviruses on cytokine levels remain unclear, leading to gaps in clinical practice. In this associative study, we measured 17 cytokines using a Bio-Plex assay in a meticulously curated plasma sample pool (N = 158) from pediatric kidney and liver transplant recipients over a one-year follow-up period. The samples included virus-negative and virus-positive cases, either individually or in combination, along with episodes of graft rejection. We observed that the elevation of IL-4, IL-8, and IL-10 correlated with graft rejection. These cytokines were elevated in samples where HCMV or HHV6 were detected alone or where EBV and HHV7 were co-detected. Interestingly, latent EBV, when detected independently, exhibited an immunomodulatory effect by downregulating cytokine levels. However, in co-detection scenarios with β-herpesviruses, EBV transitioned to a lytic state, also associating with heightened cytokinemia and graft rejection. These findings highlight the complex interactions between the immune response and herpesviruses in transplant recipients. The study advocates for enhanced monitoring of not only EBV and HCMV but also HHV6 and HHV7, providing valuable insights for improved risk assessment and targeted interventions in pediatric SOT recipients.

## 1. Introduction

The β-herpesviruses, which include cytomegalovirus (HCMV), human herpesvirus 6A (HHV6A), 6B (HHV6B), and 7 (HHV7), as well as the γ-herpesviruses, Epstein–Barr virus (EBV) and Kaposi sarcoma-associated virus (KSHV), are human viruses known for causing lifelong persistent infections. Particularly, β- and γ-herpesviruses target immune cells for infection, establishing host cell lifelong reservoirs in differentiated lymphoid and myeloid cells or hematopoietic progenitors.

A significant proportion of the global population carries at least three of these herpesviruses, which is a prevalence largely attributed to their biphasic life cycle, encompassing latent and lytic phases. The latent phase is characterized by low to no expression of viral genes, enabling evasion of the immune system and persistence in the host. In contrast, during the lytic phase, there is a heightened expression of viral genes leading to the production of new viral infectious particles [1]. The transition from the latent to the lytic phase is known as reactivation. While the majority of infected hosts remain asymptomatic, in cases of associated diseases, the reactivation of these herpesviruses is typically observed and measured as detectable viral loads in peripheral blood.

In individuals with compromised immune systems, β- and γ-herpesvirus emerge as a substantial contributor to morbidity and mortality, as exemplified in solid organ transplant recipients undergoing pharmacologic immunosuppression. The intersection of the need for immunosuppression, with consequently herpesvirus reactivation, jeopardizes the success of the transplant, leaving patients vulnerable to clinical complications, such as organ rejection, post-transplant lymphoproliferative syndrome (PTLD) and HCMV-disease [2,3,4,5,6]. This delicate balance requires optimal clinical management with a focus on the monitoring of viral loads and the implementation of preemptive strategies to mitigate the impact of the herpesviruses.

In addition to their individual effects, the simultaneous detection of multiple herpesviruses has been reported in transplanted patients with some studies supporting an association between co-detection and worse clinical outcomes [7]. The tropism for immune cells and reliance on similar mechanisms for reactivation support the notion that β- and γ-herpesviruses mutually influence their biological cycles, collectively impacting the survival of the transplanted organ and the transplanted patient [7]. Cytokines are likely important mediators of mutual connection, since all β- and γ-herpesviruses have evolved immunomodulatory genes not only to block antagonizing host responses but also to influence the activation, survival, differentiation, and expansion of the immune cells that act as reservoirs for their persistent infections. Furthermore, β- and γ-herpesviruses encode their own set of functional homologs of immune-related genes, including virokines [8,9,10].

A previous analysis of this pediatric cohort revealed associations between specific herpesviruses and rejection. Notably, HCMV and HHV6 alone appeared to be associated with rejection, while EBV contributed to rejection in co-detection events, suggesting interactions between EBV and the β-herpesviruses. In this study, searching for potential channels of herpesvirus interactions, we analyzed the concentrations of 17 cytokines in the plasma of post-transplant patients over a one-year follow-up period. We correlated these cytokine concentrations with the loads of β- and γ-herpesviruses and with graft rejection. This investigation aims to deepen our understanding of the intricate relationship between immunosuppression, herpesvirus infection, and clinical outcomes in solid organ transplant recipients.

## 2. Materials and Methods

### 2.1. Patients and Clinical Samples

**Figure 1:**
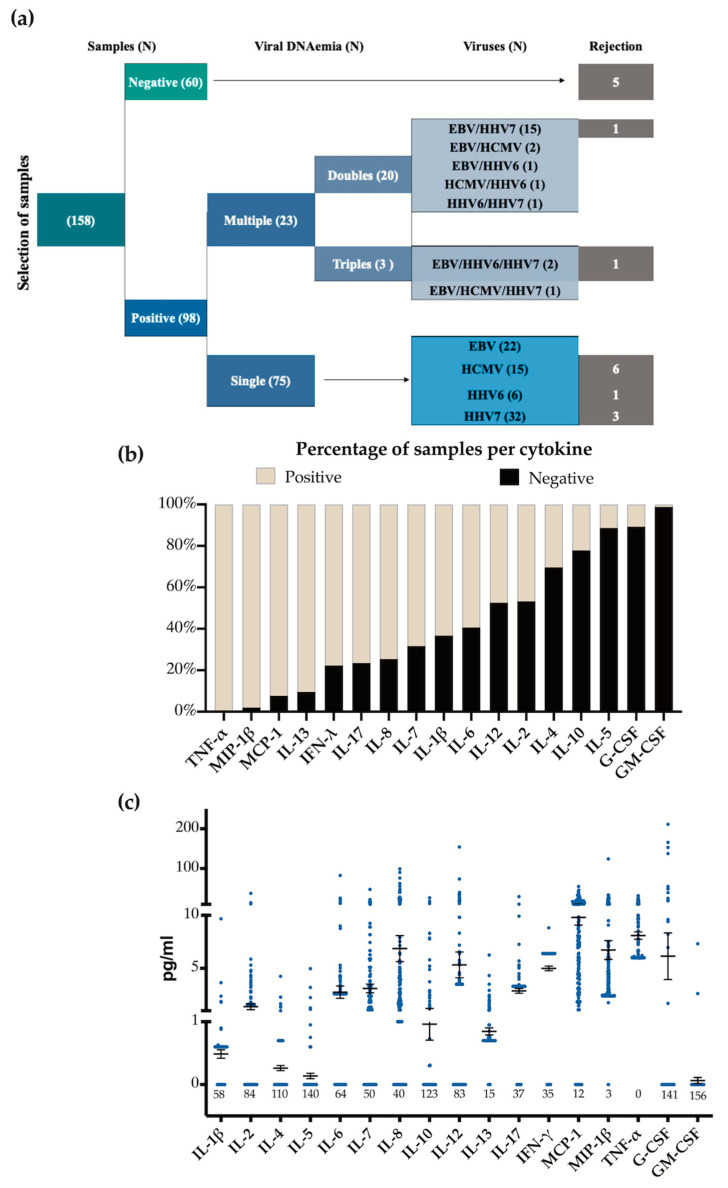
Selection of samples for cytokine analysis. (**a**) Depiction of the plasma samples analyzed. The Venn diagram shows the positive DNAemia by virus. Detection of viruses in the original cohort was as follows: HHV7 = 39%, EBV = 30%, HCMV = 20% and HHV6 = 11%. We tried to preserve this proportions in this subset of samples. (**b**) Percentage of positive (beige) and negative (black) samples for each cytokine analyzed. The numbers below are the cytokine negative samples. (**c**) Concentration for each cytokine among samples showing the mean and standard error.

**Figure A1:**
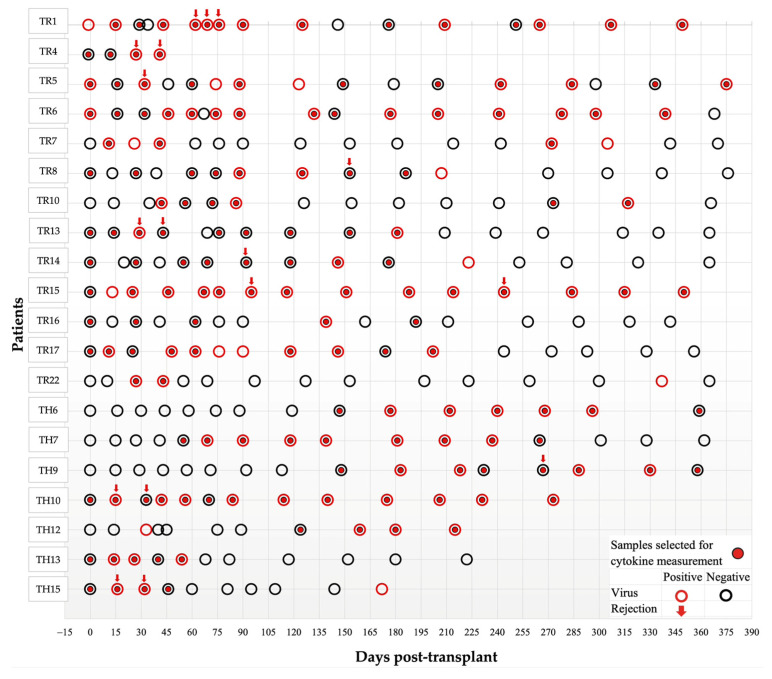
Timeline of the patients’ follow-up. The timeline indicates the samples that were positive for viral detection (red circles) and the rejection episodes (red arrows). Samples with a red fill are those taken for cytokine analysis. All analyzed samples were positive for at least one cytokine. The first 13 patients with code “TR” followed by the patient number (TR1, TR4, TR5, TR6, TR7, TR8, TR10, TR13, TR14, TR15, TR16, TR17 and TR22) were patients who underwent renal transplantation. The last 7 patients with code “TH” followed by the patient number (TH6, TH7, TH9, TH10, TH12, TH13 and TH5) were patients who underwent liver transplantation. In both Table A1 and Figure A1, renal transplant (TR), hepatic transplant (TH).

We collected a total of 158 blood samples from a cohort comprising 20 pediatric patients who underwent liver or kidney organ transplantation. These samples were carefully selected from a larger pool of 495 samples gathered over a one-year follow-up period, involving 34 post-transplant patients, 22 with renal and 12 with liver transplantation [11]. During the initial three months post-transplantation, we collected blood samples every two weeks, reducing the frequency to once per month thereafter. In a prior report, we detailed the viral DNAemia of β-herpesvirus and EBV in the leukocyte and plasma fraction of the 495 blood samples [11]. Throughout the follow-up period, ten patients experienced episodes of acute graft rejection, equivalent to 17 rejection samples, with 82% of these rejection episodes coinciding with an episode of viremia. No episode of PTLD was reported in this cohort during the follow-up. Our qPCR does not distinguish between HHV6A and HHV6B, and we will be collectively referring to these viruses as HHV6. We also analyzed the presence of KSHV, but no sample was positive for KSHV infection/detection. In the Results section, see Figure 1a for a flow chart of the samples taken for cytokine analysis; Appendix A, Figure A1 for the timeline of data collection illustrating the timepoints of viral positivity and graft rejection; and Appendix A, Table A1 for the EBV and HCMV donor and recipient serology.

As stated in our previous publication, this study was approved by the Ethical, Biosecurity and Scientific review boards of the Children’s Hospital of Mexico Federico Gómez (Registry HIM-2016-021). Graft rejection was diagnosed from clinical, laboratory and histopathological data, following the Banff global consensus classification. Prior to sample collection, patients and their parents/guardians were informed about the nature of the study, and those who were willing to participate signed a letter of consent (parents/guardians) and a letter of assent (children older than 10 years). Children with incomplete follow-up or suffering hyperacute graft rejection the first days after transplantation were excluded from the study. All enrolled patients were treated according to the ethical guidelines of our institution [11].

### 2.2. Immunoassay

We utilized 200 µL of plasma to determine the presence and concentration of cytokines IL-1β, IL-2, IL-4, IL-5, IL-6, IL-7, IL-8, IL-10, IL-12, IL-13, IL-17A, IFN-γ (interferon-γ), TNFα (tumor necrosis factor-α), MCP-1 (monocyte chemoattractant protein 1), MIP1-β (macrophage inflammatory protein 1β), G-CSF (granulocyte-colony stimulating factor) and GM-CSF (granulocyte/macrophage-colony stimulating factor) through multiplex immunoassays. We used the kit Bio-Plex Pro™ Human Cytokine 17-plex (BioRad, Hercules, CA, USA) and the Bio-Plex 200 Systems, following the manufacturer’s instructions throughout the entire process.

### 2.3. Viral Detection in Clinical Samples

Viral detection was performed using an in-house multiplex qPCR that simultaneously detects and quantifies beta and gamma human herpesviruses as previously reported [11]. In this earlier study, blood samples were fractionated into cellular components and plasma, and detection was carried out in both compartments. Since herpesviruses exhibit a bipartite life cycle oscillating between latent and lytic phases, viral loads found in the cell fraction were considered more indicative of latency, while viral loads found in plasma were considered more indicative of an active lytic cycle. It is important to highlight that before the extraction of plasma DNA, a DNase treatment step was performed to avoid quantifying viral DNA from broken cells.

### 2.4. Statistical Analysis

As we did not observe a significant difference in cytokine levels between patients who underwent kidney or liver transplants, we combined all data for statistical robustness. Forty-six percent of the cytokine-positive samples were below their technical limit of detection (LOD); to facilitate the statistical analyze of these cytokines, we used the substitution method to maintain statistical rigor [12]. This method allows the analysis of data below the LOD, which is also called censored data. The most common and easiest strategy is a simple substitution in which censored values are either replaced by zero, replaced by a fraction of the detection limit (usually 1/2 or 1/√2), or replaced with the LOD itself. After testing the substitution with zero and the LOD, and finding no differences, we opted to use each cytokine’s specific LOD to substitute the censored data (Table 1). Using substitution methods for values below the LOD can potentially distort estimates and statistical tests, particularly for cytokines with an elevated proportion of samples under the LOD. While we believe our approach is sound and follows established practices, we acknowledge this potential limitation.

**Figure A2:**
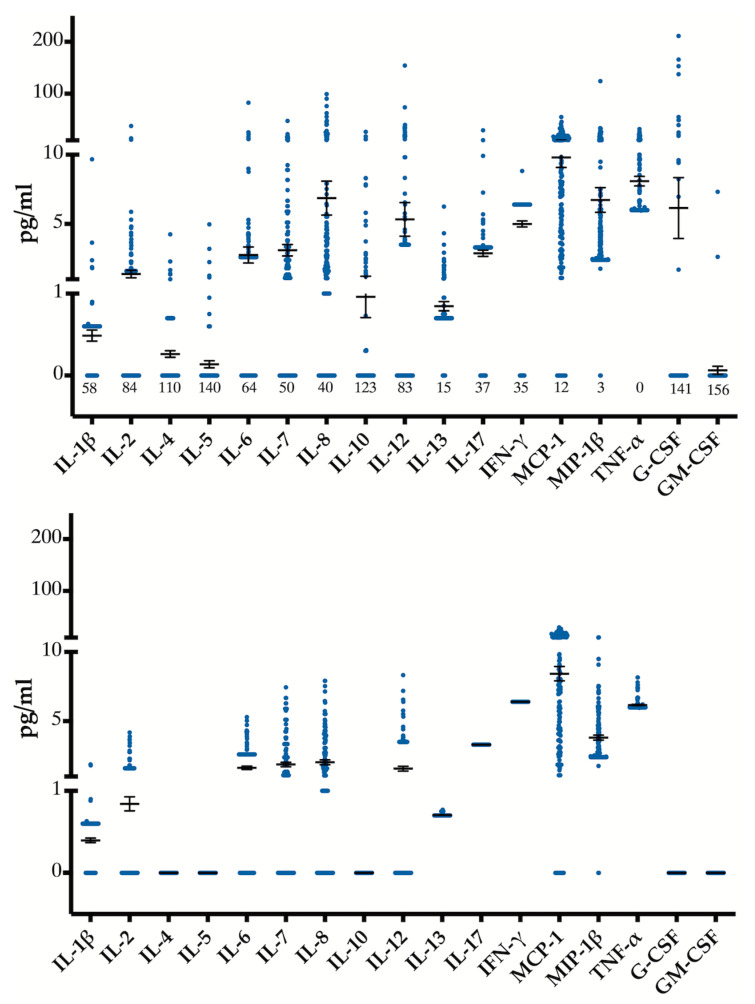
Scatter dot plots; the first is the distribution of the raw data, the second is the distribution of the clean data after the ROUT test.

Using Mann–Whitney t-tests, we compared the concentrations for each cytokine between groups, for instance, samples with positive viral DNAemia versus those negative for viral DNAemia; or samples positive for rejection versus those negative for rejection. We used the Kruskal–Wallis test to compare the cytokine concentrations among multiple groups, such as multiple, single and no viral DNAemia. Outliers were removed using a ROUT test with a Q = 1% in all comparative analyses (Figure A2). Correlation analyses were performed using Spearman tests.

We employed relative risk (RR) analyses to quantify the association between the presence of specific cytokines and the outcomes of interest, namely rejection and the detection of herpesviruses. The RR provides a measure of the strength of association between an exposure (in this case, cytokines or viral DNAemia) and an outcome (rejection or presence of a herpesvirus) (Appendix B).

Heat maps were generated to illustrate the disparities between the means of the analyzed groups. These differences were normalized to percentages, and the colorimetric scale was adjusted to reflect negative or downregulated values in blue, while upregulated or positive values were represented in yellow. This color scheme was in reference to values found in the samples negative to the variables analyzed, serving as the basal reference. We used GraphPad Prism 9 software to construct graphs and visualize data.

## 3. Results

### 3.1. TNF-α, MIP-1β, MCP-1 and IL-13 Are Elevated in the Plasma of Post-Transplant Patients

We carefully selected 158 blood samples from a larger cohort of pediatric patients who underwent liver or kidney organ transplantation (Figure 1a). The samples selected included all different variables mirroring the proportions found in the complete cohort: 98 tested positive for viral DNAemia of at least one of the herpesviruses, which is suggestive of exacerbated infection. Within this group, 23 exhibited a co-detection of multiple herpesviruses, while 75 samples showed single-virus positivity. The remaining 60 samples tested were negative for viral DNAemia. We also included in our analysis 17 samples collected during acute rejection episodes, of which five were negative to virus detection, and 12 coincided with the detection of one or more of the herpesviruses, while 55 samples were negative to rejection and viral DNAemia. All rejection episodes were T cell mediated, and graft rejection was diagnosed according to the Banff global consensus classification [13]. Table 2 shows the demographic and clinical data of this subset of patients.

We measured 17 different cytokines in the plasma of the selected samples; each of these samples tested positive for at least one of the cytokines. Figure 1b and Table 3 show the number of samples that tested positive and negative for each cytokine. TNF-α, MIP-1β, MCP-1, and IL-13 were detected in more than 90% of the samples, IFN-γ, IL-17, IL-8, IL-7, IL-1β, and IL-6 were positive in approximately 50% to 80% of the samples, and the remaining cytokines were found in fewer than 50% of the samples. Figure 1c shows the values found for each cytokine. Table S1 shows all the values found for cytokine detection and viral DNAemia in all analyzed samples.

### 3.2. Elevated Cytokines Are Preferentially Found in Samples with Multiple Viral Detection

**Figure 2:**
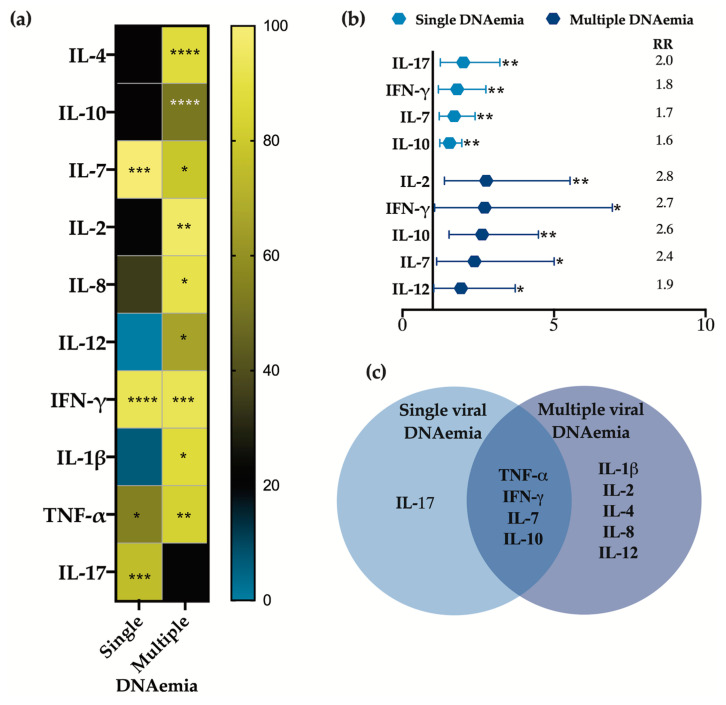
Comparison of the cytokine concentration in samples with or without viral DNAemia. (**a**) Heat map displaying the percentage increase in cytokine concentrations observed in samples with single and multiple DNAemia compared with samples negative for viral detection. The scale of cytokine expression change in percentages is shown on the right. (**b**) Forest plot representing a qualitative analysis of relative risk for cytokine positivity in single and multiple viral DNAemia samples. (**c**) Venn diagram summarizing the cytokines that exhibit differential increases in both single and multiple viral DNAemia samples. Significant values * *p* < 0.1, ** *p* < 0.01, *** *p* < 0.001 and **** *p* < 0.0001.

We compared cytokine concentrations in samples with single viral DNAemia, multiple viral DNAemia, and without DNAemia. Significant increases in the levels of TNF-α, IFN-γ, IL-17, IL-12, IL-8, IL-7, IL-2, IL-1β, IL-4, and IL-10 were observed in samples with viral detection, whether single or multiple, compared with those without viral detection. Generally, greater increases in cytokine levels were seen in samples positive for more than one herpesvirus, except for IL-7, IL-17, and IFN-γ, which were more elevated in samples with single viral DNAemia. IL-4 and IL-10 were only detected in samples with multiple DNAemia and were never detected in virus-negative or single-virus detection cases (Figure 2a). Cytokines MIP-1β, IL-13, IL-6, IL-5, G-CSF, and GM-CSF did not show any differences between sample groups and are therefore not presented.

We conducted a qualitative risk analysis considering only the frequencies at which each cytokine tested positive or negative in relation to viral DNAemia. Our results indicated that cytokines IL-2, IFN-γ, IL-10, IL-7, IL-12, and IL-17 were 1.6 to 2.8 times more frequently detected in samples with positive viral DNAemia, with a higher representation observed in cases with multiple viral DNAemia, which is consistent with the quantitative analysis (Figure 2b). Figure 2c presents a Venn diagram to summarize the quantitative and qualitative results. 

### 3.3. Elevated Cytokines Levels Correlate with Detection of the β-Herpesviruses

**Figure 3:**
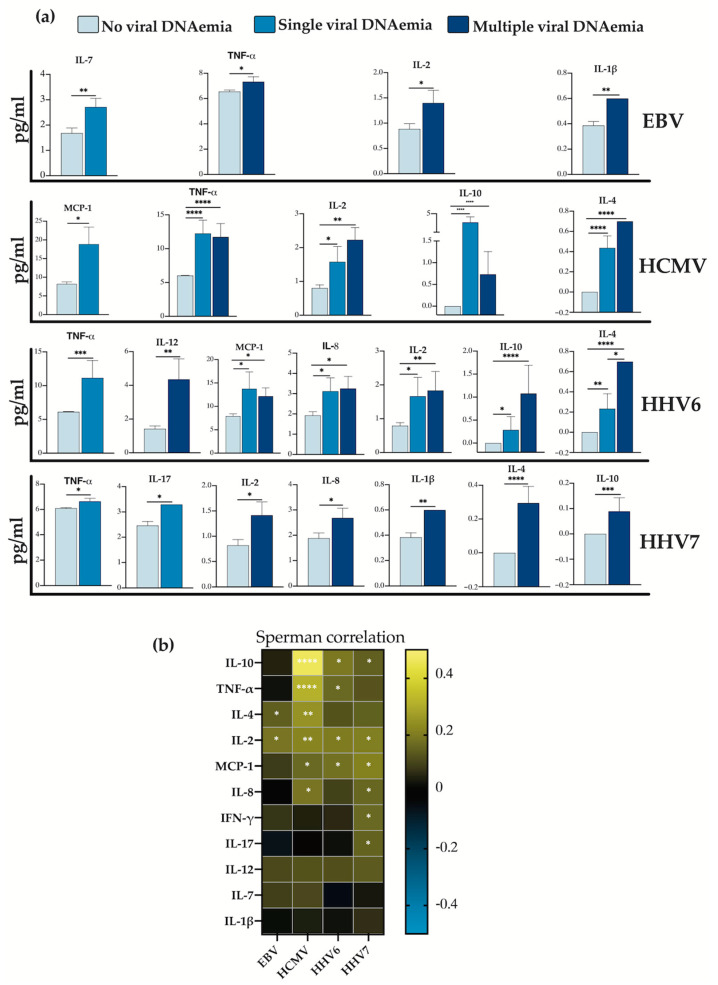
Virus-specific association with elevated cytokines. (**a**) Bar graphs, representing comparisons of the concentrations of each cytokine, between viral DNAemia-negative samples vs. samples with single and multiple viral DNAemia by virus. (**b**) Correlation matrix between the loads of each virus analyzed and the concentration of each cytokine. Significant values * *p* < 0.1, ** *p* < 0.01, *** *p* < 0.001 and **** *p* < 0.0001.

We assessed which cytokines were elevated in samples positive for each virus both in single and multiple detection events (Figure 3a). Notably, in the case of HCMV and HHV6, cytokine levels were increased in both conditions. In contrast, for EBV and HHV7, most cytokines were elevated when these viruses were co-detected with other herpesviruses. These findings suggest that HCMV and HHV6 alone can lead to high cytokine levels, whereas EBV and HHV7 appear to rely on co-detection with other herpesviruses.

To explore the connection between viral loads and cytokine concentrations, we conducted a Spearman correlation test. We observed significant but generally low to moderate positive correlations, ranging from 0.16 to 0.45 (Figure 3b). Specifically, we observed a significant correlation between the concentration of two cytokines and EBV load, four cytokines and HHV6 load, and six cytokines and either HCMV or HHV7 loads. Collectively, our findings support the notion that the detection of herpesviruses is associated with cytokine levels in post-transplant patients, particularly when multiple herpesviruses are detected. Notably, the β-herpesviruses (HCMV, HHV6 and HHV7) seem to exert a more potent influence on cytokine levels in these patients.

### 3.4. Cytokines IL-4, IL-8 and IL-10 Significantly Increase in Patients with Graft Rejection

**Figure 4:**
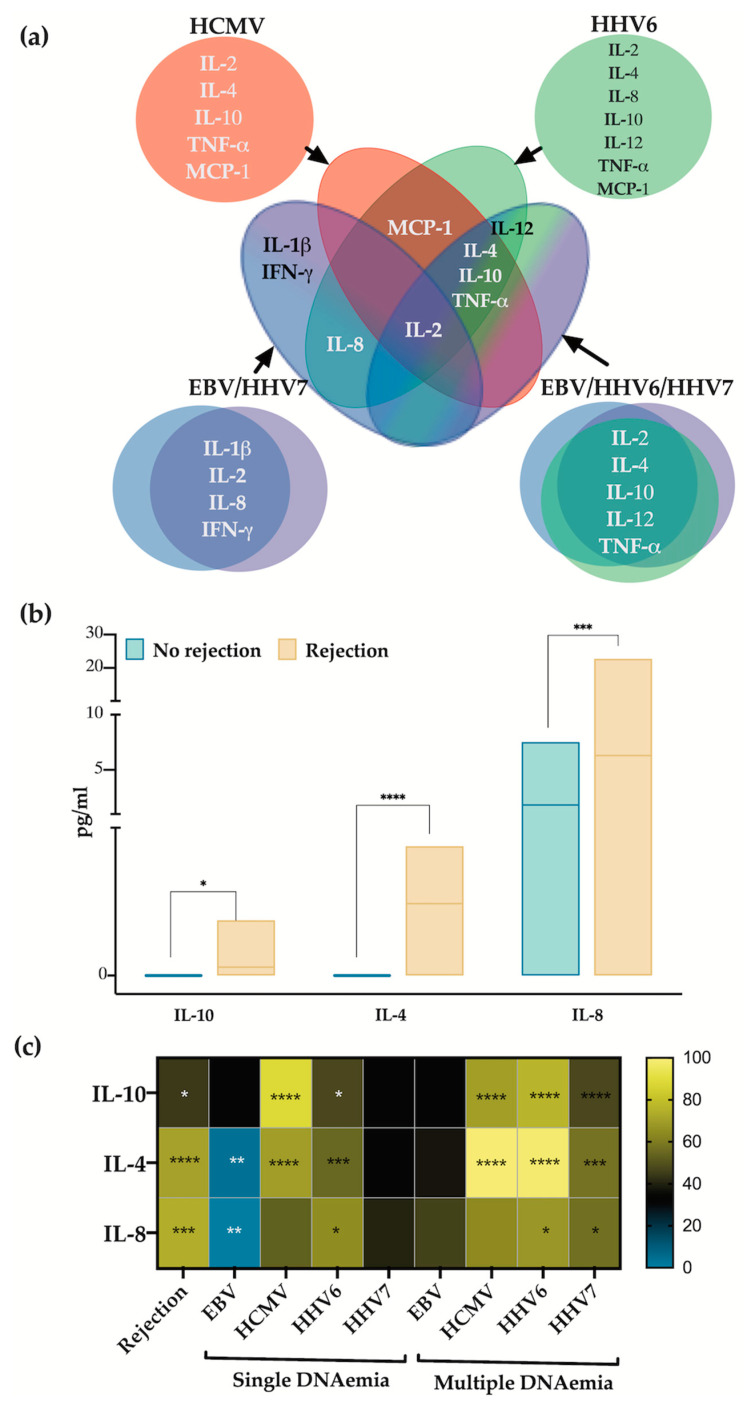
Differentially upregulated cytokines in rejection. (**a**) Venn diagram summarizes the differentially upregulated cytokines in samples with viral DNAemia by virus. (**b**) Bar graph showing the upregulated cytokines in rejection-positive samples. Kruskal–Wallis nonparametric test. (**c**) Heat map representing the comparison of cytokines between samples without viral DNAemia vs. column 1—rejection positive samples, 2—samples positive only for EBV, 3—samples positive only for HCMV, 4—samples positive only for HHV6, 5—samples positive only for HHV7, 6—samples positive for EBV plus other herpesvirus(es), 7—samples positive for HCMV plus other herpesvirus(es), 8—samples positive for HHV6 plus other herpesvirus(es), 9—samples positive for HHV7 plus other herpesvirus(es). Significant values * *p* < 0.1, ** *p* < 0.01, *** *p* < 0.001 and **** *p* < 0.0001.

In our previous report, we established an association between herpesvirus DNAemia and graft rejection in this post-transplant patient cohort [11]. We identified two viruses in single-detection (HCMV and HHV6) and two mixes of co-detected viruses (EBV/HHV7 and EBV/HHV6/HHV7) associated with graft rejection. In this study, we aimed to explore whether, within the studied cytokines, we could identify an association with the herpesviruses and/or with graft rejection. We first compiled the cytokines significantly elevated in the four conditions described above: HCMV and HHV6 in single DNAemia and EBV/HHV7 and EBV/HHV6/HHV7 co-detections (Figure 4a). Various cytokine patterns were observed; for instance, IL-2 was upregulated in all four viral detection conditions, while IL-4, IL-10, MCP-1, and TNF-α were upregulated when HCMV or HHV6 were single-detected. Conditions of EBV/HHV7 and EBV/HHV6/HHV7 co-detection did not exclusively share any cytokine, but HHV6 and EBV/HHV7 shared IL-8 upregulation.

We conducted a quantitative analysis to compare cytokine concentrations in samples associated with rejection to those without rejection. This analysis revealed a significant increase in the levels of IL-4, IL-8, and IL-10 in the rejection-positive samples (Figure 4b). We created a heat-map of the concentrations of these three cytokines with respect to the herpesviruses either in single or multiple detection (Figure 4c). IL-4 and IL-10 were consistently elevated in samples with HCMV and HHV6 detection: both alone or multiple. HHV7-positive samples also had elevated levels of these cytokines but only in multiple detection. IL-8 was elevated in samples where HHV6 was single-detected or in multiple detection events of both HHV6 and HHV7. On the contrary, EBV appeared to downregulate IL-4 and IL-8 as single detection. The analysis displayed in Figure 4c aligns with the Venn diagrams of Figure 4a, implying a strong correlation between HCMV and HHV6 detection with the upregulation of IL-4 and IL-10, and with HHV6 also correlating with IL-8. Meanwhile, detection conditions that do not involve any of these viruses (conditions with elevated EBV and HHV7) only relate to transplant rejection through IL-8. These findings suggest a role for these cytokines as mediators of virus-induced graft rejection following transplantation, with the β-herpesviruses, particularly HCMV and HHV6, identified as the main triggers of their upregulation.

### 3.5. Detection of EBV Associates with an Immunomodulatory Effect

**Figure 5:**
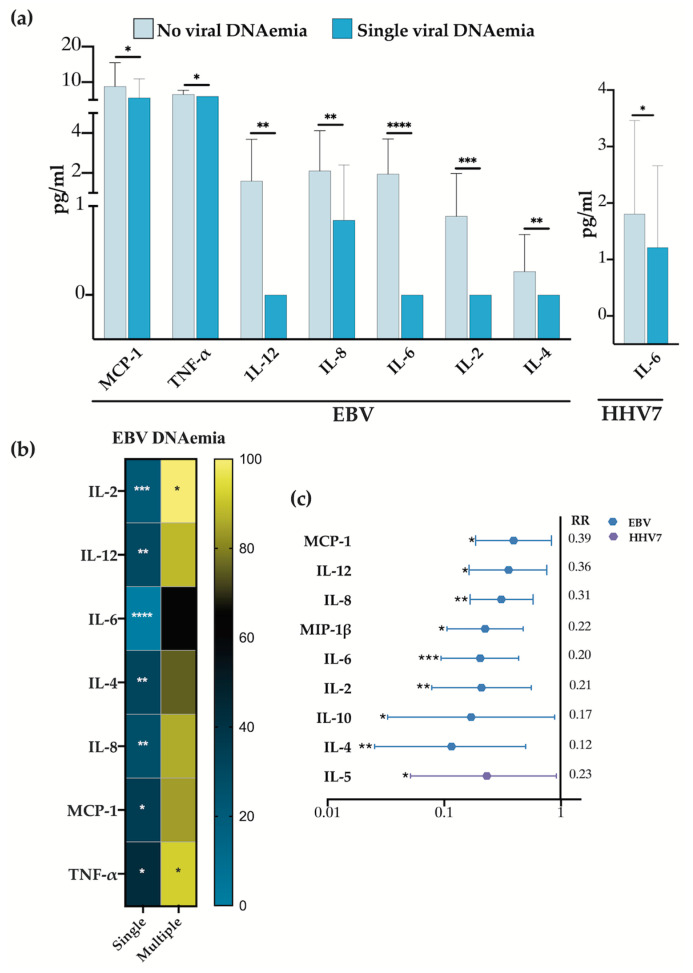
Downregulation of cytokine levels by EBV when comparing viral DNAemia-negative vs. viral DNAemia-positive samples. (**a**) Bar graph showing those cytokines decreased when comparing their concentrations between EBV DNAemia-negative vs. single EBV DNAemia samples. (**b**) Heat map showing the percentage of decrease observed between cytokine concentrations in the negative vs. positive samples both in single and multiple DNAemia. The downregulated and upregulated cytokines are shown in blue and yellow, respectively. (**c**) Forest plot of a relative risk qualitative analysis representing the protective effect yielded by EBV DNAemia. Significant values * *p* < 0.1, ** *p* < 0.01, *** *p* < 0.001 and **** *p* < 0.0001.

A comparison of cytokine concentrations across the different sample groups found that MCP-1, TNF-α, IL-12, IL-8, IL-6, IL-2, and IL-4 exhibited notably lower levels in samples with single EBV detection compared with samples where no herpesvirus was detected (Figure 5a). These findings suggest a potential immunomodulatory role for EBV. The only other instance of a cytokine exhibiting decreased concentration in samples with a single DNAemia was IL-6 and HHV7 (Figure 5a). To visualize the normalized mean difference in percent in cytokine concentrations, we generated a heat map comparing samples where herpesviruses were not detected to those with sole EBV detection (Figure 5b). The heat map illustrates how this immunomodulatory pattern is disrupted when other herpesviruses are co-detected alongside EBV, leading to increased cytokine concentrations across the board.

To provide further insights between the link of EBV detection and cytokines levels, we conducted a qualitative risk analysis similar to the one presented in Figure 2c. This analysis revealed that MCP-1, IL-12, IL-8, MIP-1β, IL-6, IL-2, IL-10, and IL-4 were less frequently observed in samples with EBV-positive single viral DNAemia compared with samples without viral detection (Figure 5c). Therefore, both the qualitative and quantitative analyses support an immunomodulatory role for EBV with both analyses showing high correlation. The sole other exception of a cytokine showing significantly lower levels was IL-5 in single- versus no-HHV7 detection (Figure 5c). However, HHV7 lacked consistency between the quantitative and qualitative risk analyses.

### 3.6. The β-Herpesviruses Appear to Reverse the EBV Immunomodulatory Effect

We tested the hypothesis that the β-herpesviruses may trigger the reactivation of EBV. In our previous analysis [11], we separated blood samples into cell and plasma fractions, reasoning that EBV detection in the cell fraction would be indicative of latent infection, while detection in the plasma fraction would be indicative of lytic infection and, consequently, of viral reactivation. We explored whether the detection of other herpesviruses alongside EBV altered the fraction where EBV was detected and whether this switch influenced the levels of the cytokines under study. This analysis aimed to provide context for understanding why EBV detection alone was associated with low cytokine levels, whereas this effect changed in cases of multiple viral detections.

**Figure 6:**
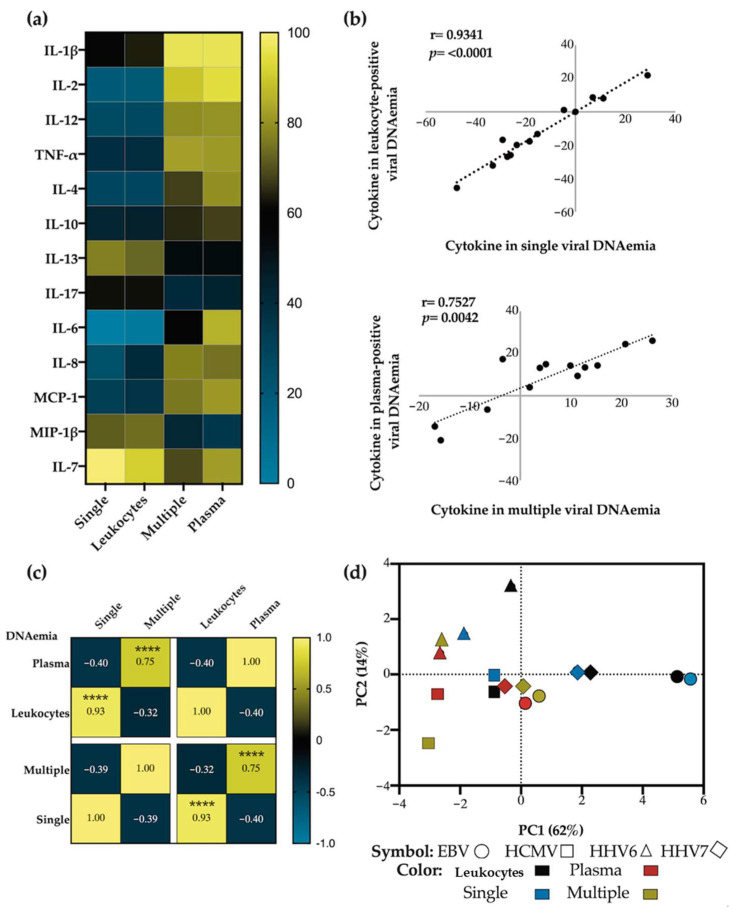
Correlation analysis of EBV detection and cytokine concentration. (**a**) Heat map representing the comparison of cytokines between samples without viral DNAemia versus four groups: column (1) EBV positive samples in single detection; (2) EBV positive samples detected in the leukocyte fraction, (3) EBV positive samples in co-detection with other herpesvirus, and (4) EBV positive samples detected in the plasma fraction. The increase in cytokines is shown in yellow and the decrease in blue. (**b**) Linear regression plots showing the correlation between cytokines levels present in samples with single viral DNAemia versus viral DNAemia detected in leukocytes (**upper panel**), and cytokines in multiple viral DNAemia versus viral DNAemia in plasma (**lower panel**). (**c**) Spearman correlation matrix showing the correlative indexes between the four groups analyzed. (**d**) Principal component analysis showing clusters of groups 1–4 for each herpesvirus analyzed. Significant values **** *p* < 0.0001.

We proceeded to analyze the patterns of cytokine concentration in patient samples, categorizing them into four groups: (1) samples with exclusive (single) EBV detection, (2) samples with EBV exclusively detected in leukocytes (suggestive of a latent state), (3) samples with co-detection of EBV and other β-herpesviruses (multiple), and (4) samples with EBV exclusively detected in plasma (suggestive of viral reactivation). As illustrated in Figure 6a, the heat map illustrates that cytokine concentration patterns are similar between groups 1 and 2 as well as between groups 3 and 4. These patterns align with low cytokine levels in the former two groups and high levels in the latter two groups. Linear correlation analysis confirmed this observation, revealing a strong positive correlation between groups 1 and 2 (r = 0.934; *p* < 0.0001) and between groups 3 and 4 (r = 0.7527; *p* = 0.0042) (Figure 6b). Conversely, correlations between groups 1 or 2 versus groups 3 or 4 were all negative (Figure 6c and Table 4).

**Figure A3:**
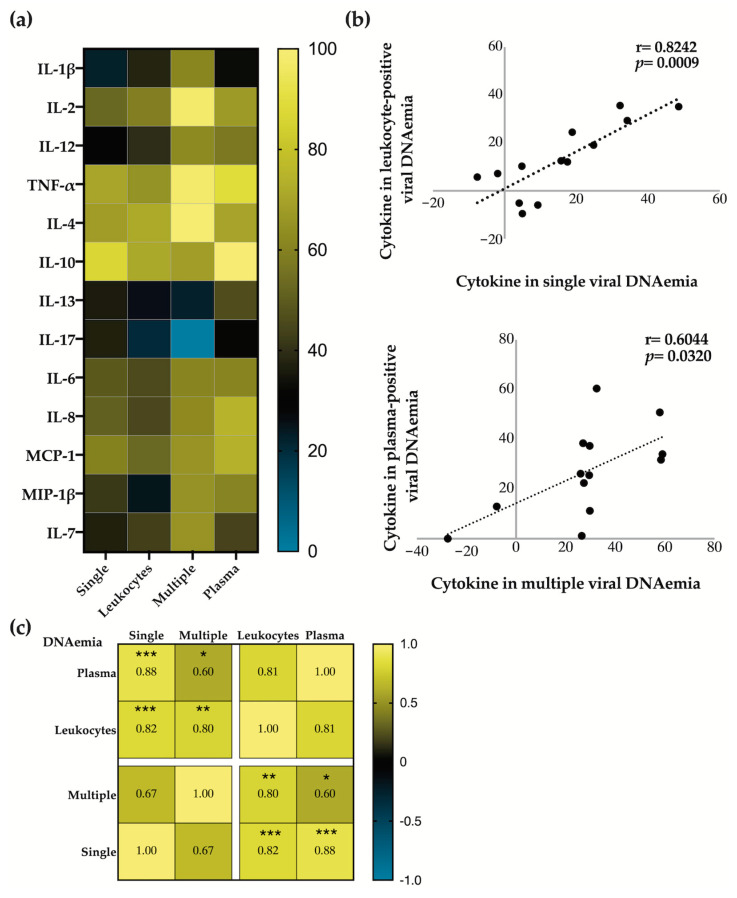
Correlation analysis of HCMV detection and cytokine concentration. (**a**) Heat map representing the comparison of cytokines between samples without viral DNAemia vs. four groups: column (1) HCMV positive samples in single detection; (2) HCMV positive samples detected in the leukocyte fraction, (3) HCMV positive samples in co-detection with other herpesvirus, and (4) HCMV positive samples detected in the plasma fraction. The increase in cytokines is shown in yellow and the decrease in blue. (**b**) Linear regression plots showing the correlation between cytokines levels present in samples with single viral DNAemia versus viral DNAemia detected in leukocytes (**upper panel**) and cytokines in multiple viral DNAemia vs. viral DNAemia in plasma (**lower panel**). (**c**) Spearman correlation matrix showing the correlative indexes between the four groups analyzed. Significant values * *p* < 0.1, ** *p* < 0.01, and *** *p* < 0.001.

**Figure A4:**
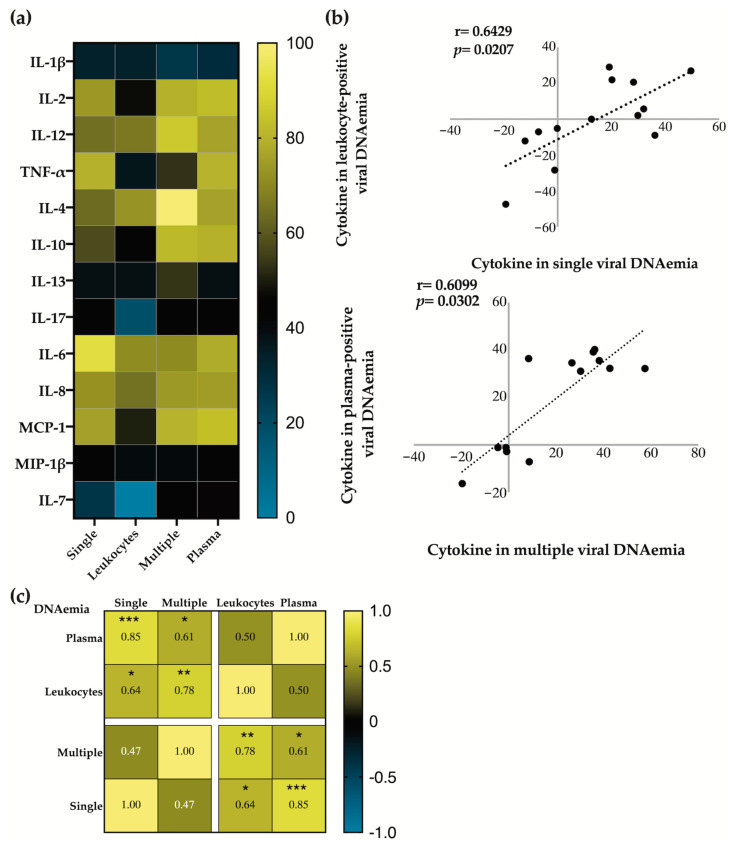
Correlation analysis of HHV6 detection and cytokine concentration. (**a**) Heat map representing the comparison of cytokines between samples without viral DNAemia vs. four groups: column (1) HHV6 positive samples in single detection; (2) HHV6 positive samples detected in the leukocyte fraction, (3) HHV6 positive samples in co-detection with other herpesvirus, and (4) HHV6 positive samples detected in the plasma fraction. The increase in cytokines is shown in yellow and the decrease in blue. (**b**) Linear regression plots showing the correlation between cytokines levels present in samples with single viral DNAemia versus viral DNAemia detected in leukocytes (**upper panel**) and cytokines in multiple viral DNAemia vs. viral DNAemia in plasma (**lower panel**). (**c**) Spearman correlation matrix showing the correlative indexes between the four groups analyzed. Significant values * *p* < 0.1, ** *p* < 0.01, and *** *p* < 0.001.

We conducted a similar analysis to the one performed for EBV with the β-herpesviruses. Interestingly, we observed with the β-herpesviruses a distinct pattern to the one observed for EBV. In the case of HCMV and HHV6, we noted positive and significant associations with cytokine concentrations regardless of the blood fraction or the presence of other viruses (Appendix A Figure A2 and Figure A3), in contrast to EBV, which showed negative correlations in the comparisons between groups: single versus plasma and multiple versus leukocytes. Therefore, only EBV in single DNAemia was associated with the downregulation of cytokine levels when viral loads were exclusively detected in the cell fraction (Table 4 provides all the statistical values). On the contrary, HCMV and HHV6 detection consistently led to heightened cytokine levels in both cellular expansions and viral reactivation as well as in single- and co-detection with other herpesviruses. HHV7 was the only virus with non-significant correlations, with only a close to significant correlation for multiple detection vs. plasma (*p* = 0.557) (Appendix A Figure A4).

**Figure A5:**
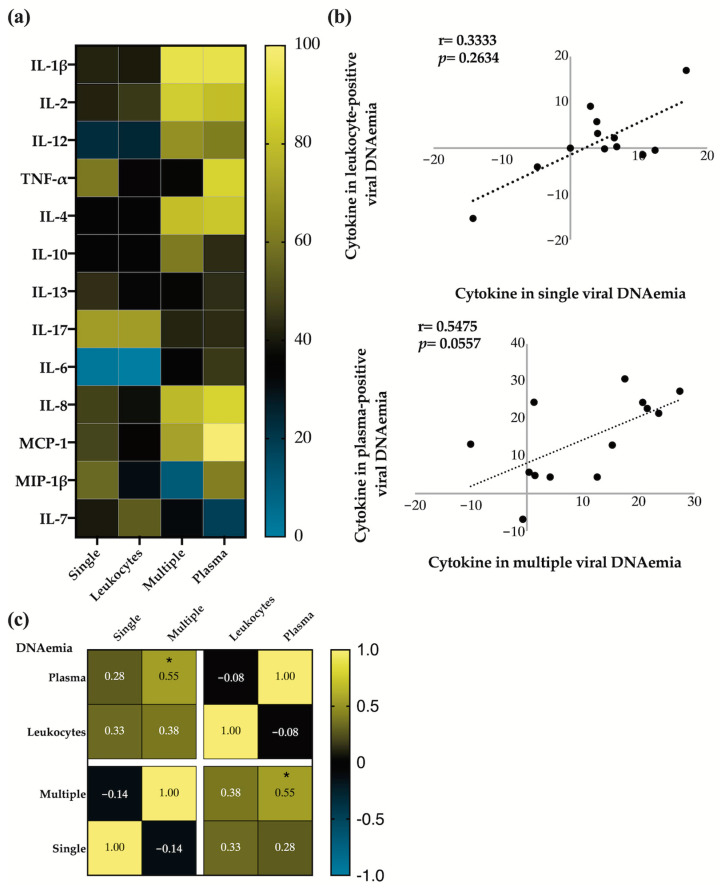
Correlation analysis of HHV7 detection and cytokine concentration. (**a**) Heat map representing the comparison of cytokines between samples without viral DNAemia vs. four groups: column (1) HHV7 positive samples in single detection; (2) HHV7 positive samples detected in the leukocyte fraction, (3) HHV7 positive samples in co-detection with other herpesvirus, and (4) HHV7 positive samples detected in the plasma fraction. The increase in cytokines is shown in yellow and the decrease in blue. (**b**) Linear regression plots showing the correlation between cytokines levels present in samples with single viral DNAemia versus viral DNAemia detected in leukocytes (**upper panel**) and cytokines in multiple viral DNAemia vs. viral DNAemia in plasma (**lower panel**). (**c**) Spearman correlation matrix showing the correlative indexes between the four groups analyzed. Significant values * *p* < 0.1.

Finally, we conducted a comparison of the four groups across all the herpesviruses using principal component analysis (PCA) (Figure 6d, Appendix A Figure A5). Once again, we noted that PC1, which explained the greatest variation in data (62%), distinctly separated EBV groups 1 and 2. EBV groups 3 and 4 appeared to localize closer to any of the other groups, including HCMV and HHV6 groups 1 to 4. The groups closest to EBV groups 1 and 2 were HHV7 groups 1 and 2, while the most distant groups were HCMV and HHV6 groups 3 and 4. This PCA analysis illustrates the unique separation of EBV groups characterized by EBV in single DNAemia, cellular fraction detection, and lower levels of cytokines. The remaining groups are mostly associated with heightened cytokine levels, and all tended to cluster in greater proximity.

Collectively, this analysis supports the idea that when EBV is detected alongside β-herpesviruses, it is in a state of lytic reactivation, whereas it appears to be in a latent state when detected alone. In the latent state, EBV associates with lower cytokine levels, whereas in reactivation, cytokine levels tend to increase. Altogether, these findings suggest that β-herpesviruses can potentially prompt EBV reactivation, leading to a loss of EBV′s immunomodulatory capacity.

## 4. Discussion

β- and γ-herpesviruses have evolved over hundreds of millions of years in close association with our immune system [14]. The capacity to alternate between latent and lytic states endows herpesviruses with a remarkable ability to achieve high fitness within hosts. Despite lifelong persistence, they are generally undetectable, indicating a homeostatic state in which latency is most probably the prevalent viral cycle. However, latency can be disturbed by molecules targeting immune cells, including cytokines, interferons, and bacterial and parasite products that stimulate pattern recognition receptors [15,16,17,18,19]. To modulate the immune system to lessen its antiviral effects and foster viral latency, approximately 30% of the β- and γ-herpesvirus genomes encode proteins or non-coding transcripts that target immune cells or immune-related processes [8,20].

There is evidence suggesting that one herpesvirus can influence the biological cycle of another herpesvirus, although such evidence is limited and has primarily been observed through in vitro experimentation. For instance, HHV6 has been shown to reactivate both EBV and KSHV; HCMV can reactivate KSHV, and HHV7 can reactivate HHV6 [21,22,23,24]. In addition to our study [11], multiple other studies support the association of elevated DNAemia of the β- and γ-herpesviruses with post-transplant complications, including EBV and HCMV [25], as well as different combinations of the β-herpesviruses [26,27,28,29,30,31,32,33,34]. In these studies, the co-detection of more than one herpesvirus is usually associated with a higher risk for an unfavorable clinical outcome. For instance, in a kidney transplant study, the co-detection of EBV and HCMV correlated with graft damage (*p* = 0.035, RR = 2.1). The detection of HHV6 and/or HHV7 often precedes HCMV detection and HCMV disease [28,34,35,36], potentially implying cross-reactivation mechanisms. In a solid organ transplant study, HCMV reduced the number of EBV-directed NK cells, increasing the risk of EBV-associated PTLD [37]. However, some studies have not found significant associations between herpesvirus co-detection and enhanced risk for rejection [38,39].

The interactions between herpesviruses appear to be significantly mediated by cytokines. For instance, it has been reported that IL-4, induced by parasites and HSV1, can reactivate γ-herpesviruses [40,41]. In vitro studies have documented that myeloid cells produce IL-1β and TNF-α in response to HHV6 infection [42], and TNF-α induces HCMV reactivation [43]. In this study, we observed an association between the cytokines IL-4, IL-8, and IL-10 with graft rejection with detection of the β-herpesviruses correlating with the upregulation of these cytokines. Notably, HCMV and HHV6 correlated with high cytokine levels whether detected individually or in conjunction with other herpesviruses. In contrast, elevated cytokine levels associated with EBV and HHV7 were predominantly observed when these viruses were co-detected with other herpesviruses. Overall, our findings suggest two potential mechanisms for graft rejection: (i) the sole deregulation of HCMV and HHV6, which alone can lead to high cytokine levels, and (ii) the simultaneous deregulation of EBV and HHV7. This latter mechanism was associated with graft rejection primarily through IL-8.

There is a high heterogeneity in the literature concerning which cytokines are important markers of graft rejection [44]. IL-4 has been associated with liver allograft rejection [45], and the blocking of IL-4 has been proposed to improve long-term grafted kidney preservation [46]. IL-8 has also been related to deterioration of the transplanted liver and proposed as a predictive marker of acute rejection in liver transplantation [47,48]. Several studies have also analyzed IL-10 levels after transplantation, yielding conflicting results. Low levels of IL-10 have been found in chronic kidney rejection [49,50], and IL-10-positive blood cells quantified through ELISPOT were found significantly diminished in acute and chronic kidney rejection [51]. On the contrary, elevated IL-10, IL-17 and IP-10 (interferon gamma-inducible protein 10) had an estimated 94% sensitivity and 97% specificity to predict graft rejection [52]. Elevated levels of IL-10 have also been proposed as a marker for an enhanced risk of HCMV disease in kidney or liver allograft recipients [53]. Importantly, EBV and HCMV secrete BCRF1 and UL111A/cmvIL-10, respectively, which are IL-10 homologous proteins that modulate the host immune system [7]. These viral IL-10s have been shown to inhibit the synthesis of several proinflammatory cytokines, such as IL-1α, IL-6, IL-12, IFN-γ, and TNF-α [9,10].

EBV appeared to display an immunomodulatory capacity, as its single detection was associated with decreased cytokine levels, including those linked with rejection. While there is abundant information about the capacity of EBV to establish an immunosuppressive environment in EBV-associated neoplasms, there is a general lack of information for other pathological conditions. For instance, EBV can downregulate the capacity of HHV6 to trigger TNF-α secretion in infected blood mononuclear cells [54]. Remarkably, the pattern of cytokine downregulation changed when EBV was co-detected with the β-herpesviruses, leading to elevated levels of the cytokines. A noteworthy observation was that EBV, when detected in isolation, was preferentially found in the cellular fraction of peripheral blood, suggesting a predominant latent state. However, in co-detection with β-herpesviruses, it was found in plasma, suggesting viral reactivation. These observations support a scenario where latent EBV can downregulate cytokine levels, and this balance is disrupted when other β-herpesviruses are upregulated. In such events, EBV is observed in plasma, hinting at a crosstalk between the herpesviruses that may trigger EBV reactivation. Conversely, β-herpesviruses were consistently associated with the upregulation of cytokines in both single and multiple detections as well as in both latent and reactivation states.

An important limitation of this study is that it is an associative study, and in this complex interplay of virus, cytokines and graft rejection, we cannot conclude which one comes first, triggering the others. For instance, IL-10 is considered the prototype of anti-inflammatory cytokines. It is conceivable that IL-10 enhanced levels may arise as a graft-protective mechanism rather than an instrument for graft damage; in other words, the upregulation of IL-10 serves as a compensatory mechanism to counterbalance graft deterioration. In support of this scenario, experimental rat models with an exogenous expression of IL-10 have demonstrated extended graft survival [55,56,57,58]. A similar protective role has been proposed for IL-4 in rat experimental models [59,60]. Another limitation of this study is that picks of viral loads and the frequency of simultaneous detections may only be reflective of the degree of host immunosuppression or the donor–receptor previous exposure to the herpesvirus of interest. We were surprised by the low levels of cytokines found in patients, but they may also be reflective of the patients′ immunosuppressive state. Collectively, the limitations of this study underscore the fundamental challenge inherent in observational research: the difficulty of inferring causality from temporal associations alone. While the findings presented here offer valuable insights and generate hypotheses regarding causality, they do not definitively establish causal relationships.

Whether they act as the cause or consequence, our study underscores the importance of monitoring of EBV, HCMV, HHV6, and HHV7, along with IL-4, IL-8, and IL-10, as markers indicating an increased risk of graft rejection during clinical follow-up after transplantation. Regrettably, current worldwide recommendations do not advocate for the consistent monitoring of HHV6 and HHV7, despite HHV6´s strong association with graft rejection. Moreover, there is a need for a standardized method of quantifying herpesviruses to establish clear thresholds of viral loads that strongly indicate unfavorable clinical outcomes. These insights are crucial for advancing our understanding of the significance of herpesvirus detection in clinical outcomes, guiding targeted therapeutic interventions, and developing refined preventive strategies. Ultimately, these efforts aim to improve the overall prognosis and enhance the quality of life for transplanted patients.

## 5. Conclusions

Latent EBV can downregulate cytokine levels, and this balance is disrupted when other β-herpesviruses are upregulated. In such events, EBV is observed in plasma, hinting at a crosstalk between the herpesviruses that may trigger EBV reactivation. Conversely, β-herpesviruses were consistently associated with the upregulation of cytokines in both single and multiple detections as well as in both latent and reactivation states. Some cytokines like IL-4, IL-8, and IL-10 can act as mediators of virus-induced graft rejection following transplantation, with the β-herpesviruses, particularly HCMV and HHV6, identified as the main triggers of their upregulation.

## Figures and Tables

**Table 1:** Limit of detection values for all cytokines tested.

Cytokine	LOD *	Cytokine	LOD *	Cytokine	LOD *	Cytokine	LOD *
IL-1β	0.6	IL-7	1.1	IL-17	3.3	GM-CSF	1.7
IL-2	1.6	IL-8	1	IFN-γ	6.4	G-CSF	2.2
IL-4	0.7	IL-10	0.3	MCP1	1.1		
IL-5	0.6	IL-12	3.5	MIP1β	2.4		
IL-6	2.6	IL-13	0.7	TNF-α	6		

* LODs: limits of detection in pgs/mL.

**Table 2:** Patients’ clinical and demographic data.

Transplant	Renal	Liver
N° of patients	13	7
Age range at transplant (median)	6–17 years (14.5)	4–8 years (4.5)
Sex		
Female	15%	57%
Male	85%	43%
Type of donor		
Diseased	54%	86%
Living	46%	14%
N° of samples (median/patient)	192 (15)	92 (13)
Pretransplant diagnosis	77% ESRD * of unknown etiology	14% Bile duct atresia
		14% Fulminant Hepatitis
	8% Focal and segmental glomerulosclerosis	14% Neonatal giant cell hepatitis
		14% Tyrosinemia
	8% ESRD secondary to **JRA hypoplasia	14% Bayler disease
		14% Alalgille syndrome
	7% Microscopic polyangiitis	14% Progressive intrahepatic family cholestasis

* ESRD: end-stage chronic kidney disease; ** JRA: juvenile rheumatoid arthritis.

**Table 3:** Positive and negative samples for each cytokine.

Cytokine	Positive	Negative	Cytokine	Positive	Negative
TNF-α	158	0	IL-6	94	64
MIP-1β	155	3	IL-12	75	83
MCP-1	146	12	IL-2	74	84
IL-13	143	15	IL-4	48	110
IFN-γ	123	35	IL-10	35	123
IL-17	121	37	IL-5	18	140
IL-8	118	40	G-CSF	17	141
IL-7	108	50	GM-CSF	2	156
IL-1β	100	58			

All cytokines were positive for at least 2 samples.

**Table 4:** Summary of the statistical values of the correlation analysis.

Virus	Comparison	r Value	*p* Value
EBV	Single (G1) vs. leukocyte (G2)	0.93	<0.0001
	Multiple (G3) vs. plasma (G4)	0.75	0.0042
	Single (G1) vs. plasma (G4)	−0.4	0.1822
	Multiple (G3) vs. leukocyte (G2)	−0.32	0.2800
HCMV	Single (G1) vs. leukocyte (G2)	0.82	0.0009
	Multiple (G3) vs. plasma (G4)	0.60	0.0320
	Single (G1) vs. plasma (G4)	0.88	0.0001
	Multiple (G3) vs. leukocyte (G2)	0.8	0.0018
HHV6	Single (G1) vs. leukocyte (G2)	0.64	0.0207
	Multiple (G3) vs. plasma (G4)	0.61	0.0302
	Single (G1) vs. plasma (G4)	0.85	0.0005
	Multiple (G3) vs. leukocyte (G2)	0.78	0.0025
HHV7	Single (G1) vs. leukocyte (G2)	0.33	0.2634
	Multiple (G3) vs. plasma (G4)	0.55	0.0557
	Single (G1) vs. plasma (G4)	0.28	0.3502
	Multiple (G3) vs. leukocyte (G2)	0.38	0.1999

## Data Availability

All relevant data related to this study is presented in main or supplementary figures and tables and in figures and tables of reference 11.

## Supplementary Materials

The following supporting information can be downloaded at https://www.mdpi.com/article/10.3390/v16071067/s1, Table S1: Cytokine concentration and viral load per sample analyzed.

## Abbreviations

EBV	Epstein–Barr virus
G-CSF	granulocyte-colony stimulating factor
GM-CSF	granulocyte/macrophage-colony stimulating factor
HCMV	human cytomegalovirus
HHV6	human herpesvirus 6
HHV7	human herpesvirus 7
IFN-γ	interferon-γ
IL	interleukin
IP-10	interferon gamma-inducible protein 10
KSHV	Kaposi sarcoma associated virus
LOD	limit of detection
MCP-1	monocyte chemoattractant protein 1
MIP1-β	macrophage inflammatory protein 1β
PCA	principal component analysis
PTLD	post-transplant lymphoproliferative syndrome
qPCR	quantitative polymerase chain reaction
RR	relative risk
TNFα	tumor necrosis factor-α

## Appendix A

**Table A1:** EBV and HCMV donor and recipient serology.

Patient ID	EBV Serology		HCMV Serology	
	Donor	Recipient	Donor	Recipient
TR1	Positive	Negative	Negative	Positive
TR4	Positive	No data	Positive	Negative
TR5	No data	No data	Positive	Positive
TR6	No data	No data	Positive	Negative
TR7	No data	No data	Positive	Positive
TR8	No data	No data	Positive	Negative
TR10	Positive	Positive	Positive	Positive
TR13	Positive	Positive	Positive	Positive
TR14	Positive	Positive	Positive	Positive
TR15	No data	No data	No data	Positive
TR16	Positive	Positive	Positive	Positive
TR17	No data	No data	Positive	Positive
TR22	Positive	Positive	Positive	Positive
TH6	Positive	Positive	Positive	Negative
TH7	Positive	Positive	Positive	Positive
TH9	Positive	Negative	Negative	Negative
TH10	Positive	Negative	Positive	Positive
TH12	Negative	Positive	Positive	Positive
TH13	Positive	Positive	Positive	Negative
TH15	Negative	No data	Positive	Negative

## Appendix B

The calculation of RR is based on a ratio of proportions, as outlined by the following equation:

$$\mathrm{RR}=\frac{a/(a+b)}{c/(c+d)}$$

where

- a represents the number of samples positive for both the cytokine and the outcome of interest (e.g., rejection or positivity to viral DNAemia);
- b is the count of samples positive for the cytokine but negative for the outcome;
- c indicates the samples negative for the cytokine but positive for the outcome; and
- d includes the samples negative for both the cytokine and the outcome.

This calculation method allows us to assess the risk of experiencing the outcome (e.g., rejection) when the exposure (cytokine) is present compared to when it is absent. Applying this analysis both to the association between cytokines and rejection, and between cytokines and each of the herpesviruses, enabled us to identify significant correlations that may influence transplant outcomes.
